# Supplementary material for: Website Investigation of Pet Weight Management-Related Information and Services Offered by Ontario Veterinary Practices
Source: Vet Sci. 2023 Nov 27;10(12):674. doi: 10.3390/vetsci10120674 (PMC10747973; doi:10.3390/vetsci10120674)
Supplement: Supplementary file 1 [file vetsci-10-00674-s001.zip › vetsci-2663777-supplementary.pdf]

veterinary sciences

MDPI

Article

Website Investigation of Pet Weight Management-Related Information and Services Offered by Ontario Veterinary Practices

Shawna Morrow, Kehan Zhang, Sarah K. Abood and Adronie Verbrugghe *

Department of Clinical Studies, Ontario Veterinary College, University of Guelph, Guelph, ON, Canada, N1G 2W1; smorrow@uoguelph.ca (S.M.); kehan@uoguelph.ca (K.Z.); sabood@uoguelph.ca (S.K.A.)

* Correspondence: averbrug@uoguelph.ca

Table S1. Website investigation survey questions and definitions.

**Survey Questions**
Practice name
Recoded ID #
Location
Alone or corporation
Species served
# of veterinarians
# of veterinary technicians
Total # of staff members listed
How many veterinarians have nutritional credentials
How many RVTs have nutritional credentials
How many veterinarians have rehabilitation credentials
How many RVTs have rehabilitation credentials
Do they have specific nutrition related staff
Do they advertise weight management service
Name of tab where weight management services are found
How many clicks to get to tab
Do they advertise nutritional counselling
Do they advertise physical therapy/ rehabilitation counselling
Do they advertise assessing patient bodyweight
Do they have veterinary therapeutic diets available
Do they have treats available
Do they have food puzzles or food measurement tools available
Do they have weight management accessories available (leashes, toys)
Body condition score form
Muscle condition score form
Diet history form
Do they have weight management or pet food information in vide form, or blogs
Written by themselves or link to other web
Easy to access from phone?
**Definitions**

Urban practice: Refers to a practice within a densely populated human settlement and corresponding infrastructure. They are made up of crowded working places and are typically categorized as cities.
Suburban practice: Refers to a practice just outside the edges of the city limits. They have a population higher than a rural area but lower than an urban area. There is a high concentration of housing with larger plots of land than urban areas but less land than rural areas. They can also be home to modest shopping centers, restaurants, and other commercial establishments.
Rural practice: Refers to a practice outside of the city and in an area with a low population density spread out over a large area. There are no crowded areas and typically consist of open fields and land. A rural area is also less likely to be home to any commercial establishments but can have necessities such as a small grocery store.
Nutrition credentials for veterinarians: Diplomate or resident of the ECVCN (European College of Veterinary and Comparative Nutrition), Diplomate or resident of the ACVN (American College of Veterinary Nutrition)
Additional nutrition credentials for veterinarians: Certificates or ongoing training in Chinese food/herb therapy (Chi Institute), PhD and MSc in pet/animal nutrition (or ongoing candidate)
Nutrition credentials for veterinary technicians: VTS Nutrition (Academy of Veterinary Nutrition Technicians).
Additional nutrition credential for veterinary technicians: Certificates or ongoing training in Chinese food/herb therapy (Chi Institute).
Physical therapy credentials for veterinarians: Diplomate or resident of the ACVSMR (American College of Veterinary Sports Medicine and Rehabilitation).
Additional Physical therapy credentials for veterinarians: Certificate or going training for: ACVAA (American College of Veterinary Anesthesia and Analgesia), CVPP (Certified Veterinary Pain Practitioner), CVA (Ceritfied Veterinary Acupuncture, Chi Institute), ACVA (The American Veterinary Chiropractic Association, CoAC(College of Animal Chiropractors), PhD and MSc in pet/animal physical therapy (or ongoing candidate)
Physical therapy credentials for veterinary technicians: Certificate or ongoing training for: The Academy of Physical Rehabilitation Veterinary Technicians, CCRT (Certified Canine Rehabilitation Therapist), CCRA (Certified Canine Rehabilitation Assistant), The Academy of Veterinary Technicians in Anesthesia and Analgesia.

Table S2. The proportion of variance in the outcome variables that can be explained by model (1) the number of veterinarians and veterinary technicians working in practice, model (2) the company status of the practice, and model (3) the location of the practice, represented by pseudo-R^2 values through Coxsnell, Nagelkerke, and McFadden models.

**Outcome variable**	**Proportion of variance determined through pseudo-R^2 values**		
	Coxsnell model	Nagelkerke model	McFadden model
**Model 1**			
Measuring current patient bodyweight	20.71%	27.64%	16.77%
Nutritional counselling	10.50%	15.54%	9.86%
Weight management service	17.29%	23.24%	13.92%
Educational material	7.41%	9.89%	5.57%
Therapeutic diets	20.21%	27.84%	17.44%
**Model 2**			
Treats & accessories	10.16%	13.66%	7.87%
**Model 3**			
Educational material	6.26%	11.05%	6.26%
